# High end GPCR design: crafted ligand design and druggability analysis using protein structure, lipophilic hotspots and explicit water networks

**DOI:** 10.1186/2193-9616-1-23

**Published:** 2013-12-20

**Authors:** Jonathan S Mason, Andrea Bortolato, Dahlia R Weiss, Francesca Deflorian, Benjamin Tehan, Fiona H Marshall

**Affiliations:** CADD/Computational Chemistry, Heptares Therapeutics Ltd, BioPark, Broadwater Road, Welwyn Garden City, AL7 3AX UK; CSO, Heptares Therapeutics Ltd, BioPark, Broadwater Road, Welwyn Garden City, AL7 3AX UK

**Keywords:** GPCR, StaR, GRID, WaterMap, WaterFLAP, metadynamics, A_2A_

## Abstract

**Purpose:**

G Protein-Coupled Receptors (GPCRs) are a large family of therapeutically important proteins and as diverse X-ray structures become available it is increasingly possible to leverage structural information for rational drug design.

We present herein approaches that use explicit water networks combined with energetic surveys of the binding site (GRID), providing an enhanced druggability and ligand design approach, with structural understanding of ligand binding, including a ‘magic’ methyl and binding site mutations, and a fast new approach to generate and score waters.

**Methods:**

The GRID program was used to identify lipophilic and hydrogen bonding hotspots. Explicit full water networks were generated and scored for (pseudo)apo structures and ligand-protein complexes using a new approach, WaterFLAP (Molecular Discovery), together with WaterMap (Schrödinger) for (pseudo)apo structures. A scoring function (MetaScore) was developed using a fast computational protocol based on several short adiabatic biased MD simulations followed by multiple short well-tempered metadynamics runs.

**Results:**

Analysis of diverse ligands binding to the adenosine A_2A_ receptor together with new structures for the δ/κ/μ opioid and CCR5 receptors confirmed the key role of lipophilic hotspots in driving ligand binding and thus design; the displacement of ‘unhappy’ waters generally found in these regions provides a key binding energy component. Complete explicit water networks could be robustly generated for protein-ligand complexes using a WaterFLAP based approach. They provide a structural understanding of structure-activity relationships such as a ‘magic methyl’ effect and with the metadynamics approach a useful estimation of the binding energy changes resulting from active site mutations.

**Conclusions:**

The promise of full structure-based drug design (SBDD) for GPCRs is now possible using a combination of advanced experimental and computational data. The conformational thermostabilisation of StaR® proteins provide the ability to easily generate biophysical screening data (binding including fragments, kinetics) and to get crystal structures with both potent and weak ligands. Explicit water networks for apo and ligand-complex structures are a critical ‘third dimension’ for SBDD and are key for understanding ligand binding energies and kinetics. GRID lipophilic hotspots are found to be key drivers for binding. In this context ‘high end’ GPCR ligand design is now enabled.

**Electronic supplementary material:**

The online version of this article (doi:10.1186/2193-9616-1-23) contains supplementary material, which is available to authorized users.

## Background

GPCRs are one of the largest families of related proteins in the human genome and as key regulators in the pathophysiology of diverse diseases are generally considered excellent targets for drug discovery (Congreve et al., 2011). X-ray structures of a diverse set of Family A GPCRs are now known, with 20 published, in mainly inactive (antagonist/inverse agonist bound) but also active (agonist bound) states, together with two recent family B structures, and one family F structure (http://gpcr.scripps.edu/). The use of fusion proteins, monoclonal antibodies and conformational thermostabilisation using the StaR® approach has enabled this enormous recent progress (Bertheleme et al., 2013;Wang et al., 2013;Hollenstein et al., 2013;Siu et al. 2013) with the latter having the advantage that a very potent ligand is not needed as part of the stabilisation. These advances in structural biology have given ‘game-changing’ insight into the binding sites of this superfamily of receptors, facilitating full structure-based drug design and providing templates for the construction of homology models (Kobilka, 2013;Mason et al., 2012). The StaR thermostabilisation process has enabled structures with multiple ligands to be obtained at Heptares for Drug Discovery projects including adenosine A_2A_ receptor (A_2A_) antagonists, muscarinic M1 agonists and dual orexin 1/2 antagonists.

In previous papers (Congreve et al., 2012;Mason et al., 2012;Langmead et al., 2012) we discussed target druggability and the SBDD of novel ligands for the adenosine receptor. Key aspects of these analyses were the water network energetics and the properties of the binding site determined by GRID (Goodford, 1985;Sciabola et al., 2010) probes, in particular the hotspots for lipophilic and hydrogen bonding groups. Regions with waters termed ‘unhappy’ (as they would prefer to be in bulk solvent, calculated using the WaterMap software) and lipophilic/hydrophobic hotspots, particularly when adjacent to hydrogen bonding hotspots, were found to be drivers for druggability, allowing the efficient design of potent ligands with good drug-like properties.

Waters are increasingly being implicated in many aspects of ligand binding (Snyder et al., 2011;Breiten et al., 2013), including kinetics (Bortolato et al., 2013;Pearlstein et al., 2013). Indeed, they can be considered to be the third dimension in understanding ligand binding and kinetics after the protein and the ligand. Water mediated interactions of ligands with receptors have always been considered important, but generally ignored when not seen directly in an X-ray structure. In GPCR-ligand binding such interactions can be critical; ideally, computational approaches would be able to create and score water networks in real-time for ligand design and binding mode analysis. We report herein results with a new fast approach based on molecular interaction fields (MIFs) that have been over many years optimised in the GRID and now FLAP/WaterFLAP software (Baroni et al., 2007;FLAP/WaterFLAP 2013).

In this report, we continue to investigate the importance of lipophilic hotspots in druggability using several new peptide- and protein-binding GPCR X-ray structures, as well as multiple diverse ligands in a single GPCR binding site. We previously highlighted how within these lipophilic regions are often found ‘unhappy’ waters (Mason et al., 2012), i.e. waters that energetically would significantly prefer to be in bulk solvent (but remain as creating a vacuum would be even less favourable). Potent GPCR ligands have been seen in X-ray structures to displace many such waters, and a druggability analysis looks for these waters occurring in pockets that have hotspots for both lipophilic and hydrogen-bonding (water probe) groups, enabling ligands with drug-like properties to be designed. We have further investigated the importance of these lipophilic hotspots to drive ligand design by analysing different series of adenosine A_2A_ antagonists that bind to different combinations of lipophilic hotspots, including to a region where the waters did not at first stand out as particularly ‘unhappy’ (WaterMap calculation).

To have available a fast approach to generate an explicit and complete water network, robust for both apo and ligand complex structures, we have investigated an alternative way of creating and scoring a water network, using the GRID water probe iteratively to fill a binding site with waters (placed in hotspots). It is also very useful for design to have a water network with explicit hydrogens to show a plausible H-bonding network. The initial network from the water hotspots can be optimized using short equilibrating molecular dynamics (MD) simulations and the waters rescored using GRID probes. To this end a new probe (CRY) was created (Bortolato et al., 2013) to bring together lipophilic and hydrophobic probes. CRY is based on GRID C1= (carbon sp^2^ probe, lipophilic) and the DRY probe (hydrophobic interactions, limited entropy term). This new probe gives the best of both in a single probe, used for scoring waters and for a broader analysis of a binding site. An important part of this approach was that the scoring of water energies would take into account an explicit network of waters as well as the ligand.

In a further investigation of the role of water networks in ligand binding we examined an interesting structure-activity relationship (SAR), the ‘magic methyl’. One recently reported that gives more than 30 fold increase in potency for a μ opioid ligand (Lunn et al., 2011) could not be investigated as structural data was not available. We thus investigated an interesting ‘magic methyl’ effect of similar magnitude (33x) in our chromone series of A_2A_ antagonists, with BioPhysical Mapping™ (BPM) data (Zhukov et al., 2011) highlighting its binding mode, to illustrate the power of lipophilic hotspots and the use of explicit water networks, e.g. in molecular dynamics.

The analysis of the water network in the active site can be important also for the understanding of site directed mutagenesis effects on ligand binding. In particular we evaluated the possibility to apply WaterFLAP to interpret BPM data (Zhukov et al., 2011;Bortolato et al., 2013). BPM is an experimental approach used to map binding site interactions with a ligand of interest. In the BPM approach additional single mutations are added to the StaR at positions that could be involved in small molecule interactions. We combined WaterFLAP with a fast protocol based on enhanced-sampling molecular dynamics (MetaScore) to estimate the effect of two binding site mutations on two small molecules antagonists. Metascore is based on 6 quick consecutive adiabatic bias (AB) MD simulations (Marchi and Ballone, 1999) and a total of 102 short well-tempered metadynamics runs (Barducci et al., 2008). ABMD was used to predict a possible ligand binding path. This method biases the system towards a given value of coordinates of the atoms in the system, in this case corresponding to the ligand-bound conformation. A harmonic bias acts only when the distance to the target bound state is bigger than its minimum value previously reached during the simulation (Marchi and Ballone, 1999). Metadynamics is an enhanced sampling algorithm within the framework of classical MD that enables efficient exploration of the multidimensional free energy surfaces of biological systems by adding a non-Markovian (history-dependent) bias to the interaction potential in the space defined by one or few collective variables (CVs). Well-tempered metadynamics is a variant of the original metadynamics algorithm that enables assessment of simulation convergence while keeping the computational effort focused on physically relevant regions of the conformational space (Barducci et al., 2008). MetaScore uses a path CV based on the ABMD binding path trajectory. In this particular case, MetaScore was able to estimate the qualitative effect of the mutation on the ligand binding dissociation constant (K_D_). This method provides a further complementary approach with WaterFLAP which is used to propose a possible role of the water network on the small molecule binding affinity.

## Methods

### Binding site analysis

Analysis and visualization of the protein binding site hotspots was completed using GRID (Goodford, 1985;Sciabola et al., 2010) energetic surveys and the resultant maps for probes of interest. The C3 (sp^3^ carbon) methyl probe was used to generate the surface of the protein active site, in terms of how close a carbon atom can be. Lipophilic hotspots were identified using the C1= aromatic/sp^2^ carbon probe or with the new probe termed CRY that combines the C1= probe with the DRY hydrophobic probe, that has an empirical entropy term. The CRY probe thus provides a more complete mapping of the lipophilic and hydrophobic hotspots in the binding site, and regions for aromatic π-π stacking as well as small lipophilic hotspots are identified. The CRY probe is used as part of the scoring of waters.

### Water network generation and scoring

As molecular dynamics studies can now be run much faster, they can be used to rapidly refine a network and provide more advanced scoring. Two approaches were used, based on the GRID/WaterFLAP and WaterMap software.

#### WaterFLAP

WaterFLAP is a new approach to generate and score water networks for both apo and ligand-complex structures (WaterFLAP software is being developed in collaboration with Molecular Discovery). It represents an extension of the FLAP software, where GRID water hotspots are used in an iterative fashion, each iteration taking into account the waters already added, to create a complete water network. This initial network is subjected to a short molecular dynamics (MD) optimisation. In the final optimized water network the waters are scored using the water and CRY probes energies in a manner that takes into account the presence of both the ligand and all the rest of the water network. This approach provides a protocol for the water network creation and scoring that is complementary to the other method used, WaterMap (2013). WaterFLAP can be applied to a protein-ligand complex in less than 2 hours on a desktop workstation including molecular dynamics optimization.

#### Water network creation

Initial placement of water was calculated by the Flapwater module in FLAP/WaterFLAP (2013) at a radius of 10 Å from the ligand. Water is placed in the most favourable positions based on the water OH2 GRID hotspots iteratively, where GRID hotspots are recalculated after each round of water placement. An energy cutoff is used, where only waters under the cutoff are considered at each iteration, and the cutoff is raised at each iteration from an initial to final cutoff energy. Flapwater was run to convergence, with an initial cutoff energy of -8 kcal/mol and a final cutoff energy of -1 kcal/mol.

To achieve a hydrogen bonded water network, the initial Flapwater output was relaxed with a short MD simulation. Simulations were run using the GROMACS (v4.6.1) software package. The protein and ligand complex was simulated in a box containing the initial waters placed by Flapwater, and solvated with an additional ~13,000 explicit water molecules using the TIP3P water model. Simulations were run in the NPT ensemble (constant number of molecules, pressure, and temperature) at 300 K using the AMBER99SB all-atom force field (Lindorff-Larsen et al., 2010). Ligand parameters were calculated using the ACPYPE software (Sousa da Silva and Vranken, 2012), also based on the GAFF force field parameters. Simulations were run for 20 ps, using a timestep of 0.002 ps, with the protein and ligand heavy atoms under positional restraints, and the water atoms free to move. The final frame of the 20 ps simulation was saved for further analysis. Waters within a 8 Å radius of the ligand were rescored after a single iteration of refinement using the water OH2 and CRY probe of the GRID program.

#### Adenosine receptor with triazine ligands

Inactive adenosine A_2A_ StaR receptor in complex with triazine 4g (PDB:3UZA), and triazine 4e (PDB:3UZC), was used as the starting structure. Ligands 4a and 4d were superimposed on the 4g ligand in structure 3UZA as the starting position. Flapwater followed by a 20 ps MD simulation (described above) was run separately for each starting structure. This method is fast, completing in < 2 hours on a single Intel 3.6 GHz cpu, with Flapwater and rescoring as the slowest steps.

### WaterMap

WaterMap (Abel et al., 2011;Beuming et al., 2012;Wang et al., 2011) is established software from Schrödinger that exploits an all atom explicit solvent molecular dynamics simulation followed by a statistical thermodynamic analysis of water clusters (hydration sites). Briefly, in WaterMap a pre-production simulation of 120 ps at 300 K is followed by a production simulation of 2 ns at 300 K in the NTP ensemble. The excess entropy is computed by numerically integrating a local expansion of spatial and orientational correlation functions. The enthalpy is computed by averaging the molecular mechanics energies of the water molecules in each hydration site over all frames of the molecule dynamics simulation. WaterMap waters that are calculated to have a significant positive free energy relative to being in bulk solvent are termed ‘unhappy’ and are coloured red in the figures. WaterMap was only used for (pseudo)apo structures in the work presented here.

### Ligand binding changes on changing structure

The adenosine A_2A_ receptor chromone ligands showed some cases of strong SAR, such as a ‘magic methyl’. To test whether water placement followed by MD simulation could differentiate strong versus weak binders, and provide a structural understanding, we modelled starting positions of ligands chromone12 and des-methylchromone12 into the high-resolution adenosine A_2A_ receptor structure (PDB:4EIY). For the more potent ligand chromone12 the position from Biophysical Mapping (Langmead et al., 2012) supported by a lower resolution X-ray structures (unpublished data) was used, with the methyl on the thiazole removed for the initial placement of the des-methyl derivative. In the starting position both ligands make a crucial hydrogen bonding interaction with key residue Asn253^6.55^. After initial water placement with WaterFLAP, MD simulation was run for 100 ps with positional restraints on the protein heavy atoms, but no positional restraints on the ligand or water positions. The MD simulation took <1 hour on 16 AMD 6386 SE CPU cores, making this a potentially relatively fast and inexpensive method to predict relative binding affinities of small changes in ligand structure.

### MetaScore

The 3D coordinates of the adenosine A_2A_ receptor in complex with 4g (PDB:3UZA) (Congreve et al., 2012) and ZM241385 (PDB:3PWH) (Dore et al., 2011) were used. The receptors have been prepared with the Protein Preparation Wizard in Maestro 9.2 (2011), hydrogen atoms were added and the H-bond network optimized through an exhaustive sampling of hydroxyl and thiol moieties, tautomeric and ionic state of His and 180° rotations of the terminal dihedral angle of amide groups of Asp and Gln. The tautomer with the hydrogen on the δ nitrogen has been considered for His278^7.43^ (superscripts refer to Ballesteros-Weinstein numbering) (Ballesteros et al., 1995). Hydrogen atoms have been energy minimized using the OPLS2005 force field. The A_2A_ StaR system used to determine the SPR measurements from which the kinetics were derived (Congreve et al., 2012;Zhukov et al., 2011) have been created for both complexes *in silico,* back mutating A277^7.42^ to the wild type residue Ser using Maestro and optimizing the side chain conformation. In a similar way the mutant L85A^1.52^ has been created.

We developed a scoring function (MetaScore) using a fast computational protocol based on several short adiabatic biased molecular dynamics simulations (Marchi and Ballone, 1999;Provasi and Filizola, 2010) followed by multiple short well-tempered metadynamics runs (Barducci et al., 2008;Provasi et al., 2009). Metascore is based on an automatic python script protocol using the molecular dynamics software GROMACS (v4.6.1), PLUMED (v1.3.0) and the PyMol API. MetaScore is composed of two stages, each divided in two steps.

*Stage 1* - binding path prediction. This is calculated once per protein-ligand complex (4 g-A_2A_ StaR and ZM241385-A_2A_ StaR).

(Step A) System creation and quick MD simulation. The ligands were manually positioned in Maestro in the extracellular side bulk solvent at about 25 Å from the final docked position. The AMBER99SB force field (Lindorff-Larsen et al., 2010) parameters were used for the protein and the GAFF force field (Wang et al., 2004) for the ligands using AM1-BCC partial charges (Jakalian et al., 2002). A triclinic box was defined with at least 20 Å of solvation layer around the system with periodic boundary conditions. The SPC water model was used and ions were added to neutralize the system (final concentration 0.01 M). Position restraints were always applied to protein Cα atoms (1000 kJ^-1^ mol^-1^ nm^-1^). Lennard-Jones and Coulomb interactions were treated with a cutoff of 1.1 nm with particle-mesh Ewald electrostatics (PME) (Darden et al., 1993). An energy minimization protocol based on 200 steps steepest-descent algorithm followed by 1000 steps conjugate gradient algorithm is applied to the system. A quick 2 ps MD is executed in the NPT ensemble using v-rescale (Bussi et al., 2007) (tau_t = 0.1 ps) for the temperature coupling to maintain the temperature of 300 K and using Berendsen (Berendsen et al., 1984) (tau_p = 0.5 ps) for the pressure coupling to maintain the pressure of 1 bar.

(Step B) Adiabatic bias MD. 6 consecutive simulations of 50 ps each were used to simulate the binding event of the ligand to the protein. The ligand target conformation (using in PLUMED the MSD TARGETED option) was the final crystallographic pose of the small molecule in the receptor. For the first simulation the initial target and kappa values were 10 Å and 1 kJ/nm^2^. After each simulation the target value was divided by 100 and the kappa multiplied by 100. For this part, the Parinello-Rhaman barostat (Parinello and Rhaman, 1981) barostat was used instead of Berendsen. 102 snapshots are at the end generated from the binding path trajectory.

*Stage 2* - Metadynamics energy evaluation of the binding path. The same binding path is used for the A_2A_ StaR, L85A and S277A mutants. For every snapshot of the 102 generated by Stage 1 the following steps are executed:

(Step A) System creation and quick MD simulation. The protein and ligand are structurally aligned to the corresponding protein and ligand in the snapshot. The same protocol of Stage 1 - Step A is executed.

(Step B) Metadynamics. We used a 2 ps well-tempered metadynamics (simulated temperature 300 K, bias factor 50, initial energy bias Gaussian height of 3 kcal/mol) using 1 path collective variable (S_PATH, Lambda = 0.6 and Sigma = 0.1) based on two reference frames: the starting unbound ligand conformation and the final protein-bound crystal pose. The Parinello-Rhaman barostat was used instead of Berendsen.

**Figure 1:**
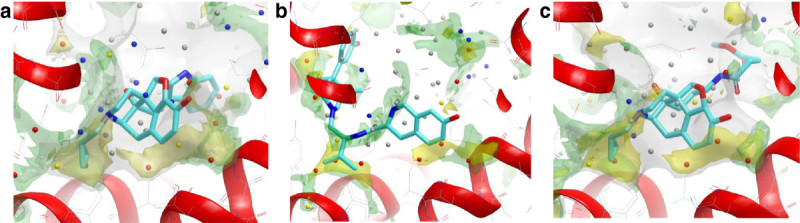
**Druggability analysis for the δ, μ and κ opioid GPCR structures. (a)** Inactive conformation of **δ** opioid with naltrindole bound (PDB:4EJ4). **(b)** Inactive conformation of **μ** opioid with β-funaltrexamine bound (PDB:4DKL). **(c)** Inactive conformation of **κ** opioid with JDTic ligand bound (PDB:4DJH). The carbon atoms of the ligands are coloured cyan. GRID maps are contoured (transparent solid) and coloured in the following manner: C1= probe (lipophilic) in yellow at –2.7 kcal/mol, water (OH2) probe in green at –6.0 kcal/mol, CH3 methyl group probe in grey at 1 kcal/mol which defines the pocket surface in terms of how close a ligand carbon atom can go. WaterMap water clusters (shown as large spheres) have been colour coded in red if predicted to have a free energy (ΔG) >3.5 kcal/mol, in yellow if their predicted ΔG is between 2.0 and 3.5 kcal/mol, in grey if ΔG is –1.0 to 2.0 kcal/mol and in blue if ΔG < –1.0 kcal/mol. All WaterMap free energy estimations are relative to bulk solvent. All figures have been made in VIDA (OpenEye) using the same ligand-protein orientation shown in Congreve et al. (2011) and Mason et al. (2012).

In Stage 2, all the 102 independent metadynamics runs explore the same energy surface corresponding to the binding event (they are writing to the same HILLS file and the PLUMED keyword RESTART is used). The final MetaScore ΔG_bind_ for the ligand is calculated as the energy difference between the bound state and the unbound state (Figure 1).

## Results and discussion

### Druggability update for GPCRs

We have extended our previous druggability analysis (Mason et al., 2012) to newer GPCRs, namely the opioid and chemokine receptor structures. In addition, the analysis of two lead discovery projects for the adenosine A_2A_ receptor highlights how lipophilic hotspots are key drivers for ligand binding and design. It is now clear that potent compounds can be designed that interact with different combinations of the possible lipophilic regions. The GRID C1= (carbon sp^2^) probe is particularly effective in the detection of lipophilic regions that are often occupied by ‘unhappy’ waters (Mason et al., 2012). These are waters that energetically would significantly prefer to be in bulk solvent but remain as creating a vacuum would be even less favourable. X-ray structures show displacement of such waters, that drives GPCR ligand potency. A druggability analysis looks for ‘unhappy’ waters occurring in pockets that have hotspots for both lipophilic and hydrogen-bonding (water probe) groups, enabling potent ligands with drug-like properties to be designed.

**Figure 2:**
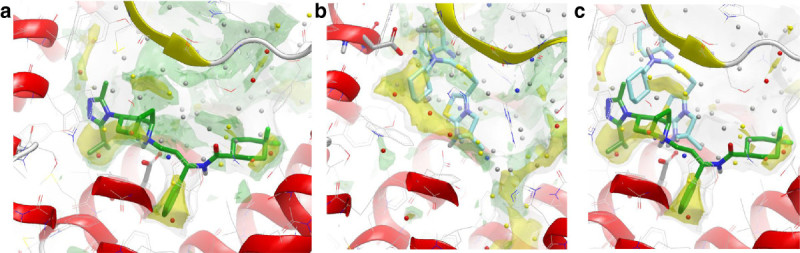
**Druggability analysis for the CCR5 chemokine GPCR structure with maraviroc bound using GRID and WaterMap. (a)** The inactive conformation of the chemokine CCR5 with maraviroc (carbon atoms in green) bound (PDB:4MBS). **(b)** Inactive CXCR4-T4L in complex with IT1t (carbon atoms in cyan) (PDB:3ODU). **(c)** For comparison, the position of the ligand IT1t from the aligned CXCR4 structure is shown in the binding site of the CCR5 structure with maraviroc bound. The GRID maps and WaterMap waters are contoured and colour coded as in Figure 1.

**Figure 3:**
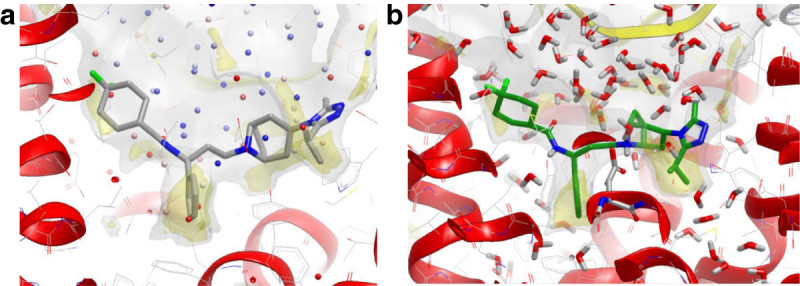
**WaterFLAP water networks for the CCR5 chemokine GPCR structure with maraviroc bound. (a)** The water network for the pseudo-apo structure from WaterFLAP post MD and rescored with the GRID water and CRY probes, coloured from red to blue for ‘unhappy’ to ‘happy’. **(b)** The water network from WaterFLAP of the complex post MD; the Glu283 interacting with the maraviroc basic nitrogen is highlighted in stick. The GRID C1= lipophilic hotspot map is shown in yellow contoured at -2.7 kcal/mol in each figure.

Several important new GPCR structures have been published since our druggability paper, including three opioid structures in the inactive form. Figure 1a-c shows the druggability analysis for the δ, μ and κ opioid GPCR structures with ligands bound. The ligands all bind deep in the pocket, with two key lipophilic hotspot interactions that contain ‘unhappy’ waters. The most recent structure is the C-C chemokine receptor type 5 (CCR5) structure (Tan et al., 2013) in complex with maraviroc (Selzentrty)(PDB:4MBS), that highlights the exciting new insights for drug design usually found with new GPCR structures. This structure reveals a chemokine binding site quite different from the previous chemokine structure, the CXCR4 structure (Wu et al., 2010). The ligand binds in an extended conformation deeper in the site, at a similar depth to many other Family A GPCRs (Figure 2a). Trp86 on helix 2 has moved to create a larger pocket deep in the site. Interestingly there are now 3 lipophilic hotspots, spread over 14 Angstroms, all covered by maraviroc; in the CXCR4-IT1t structure there was a single large lipophilic-only hotspot with the ‘unhappy’ waters higher in the site (Figure 2b and aligned with the CCR5 structure in Figure 2c). The centre lipophilic hotspot (phenyl group in maraviroc) is lipophilic only, as is the less deep single hotspot in the CXCR4-IT1t structure, but the other two lipophilic hotspots are more druggable, with adjacent water hotspots. This is shown in Figure 2a (GRID lipophilic and water hotspots in yellow and green respectively). These three key lipophilic hotspots contain ‘unhappy’ waters (shown for the WaterMap calculation on pseudo-apo structure) and provide a framework to drive the design of new ligands. Figure 3a shows the result for the same structure using the new WaterFLAP protocol resulting in a similar prediction, with the lipophilic hotspots containing the most ‘unhappy’ waters. In WaterFLAP waters are iteratively added to GRID water probe hotspots, the resulting network is optimised using MD and the final waters are rescored with GRID water and CRY probes. The WaterFLAP protocol we use, initial water placement followed by a short MD optimization of the network, is also good at producing explicit water networks for ligand-protein complexes, to aid further design etc., and the results for the maraviroc complex are shown in Figure 3b.

### Importance of lipophilic hotspots for ligand binding

**Figure 4:**
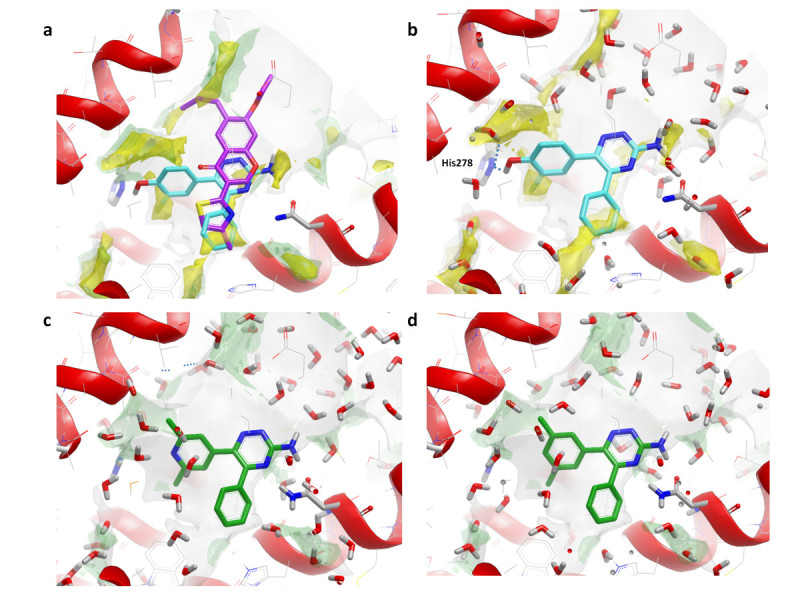
**Analysis of the binding of two potent ligand series found by virtual screening for the adenosine A**_**2A**_**receptor. (a)** The triazine **4e** (cyan carbon atoms) bound to the inactive conformation of A_2A_ (PDB:3UZC) together with a high confidence docking of the chromone12 (pKi 8.5) from Biophysical Mapping™ and X-ray data (unpublished) showing how each ligand exploits two key lipophilic sites; GRID maps with lipophilic hotspots in yellow at -2.5 kcal/mol, water hotspots in green at -6 kcal/mol. **(b)** The WaterFLAP calculated water network post MD in stick for the X-ray complex of compound **4e** with the A_2A_ receptor with the histidine H-bonding to the phenol highlighted in stick with only the GRID lipophilic hotspots shown. **(c)** The WaterFLAP calculated water network post MD in stick for the X-ray complex of compound **4g** with the A_2A_ receptor showing a good network with the pyridine; GRID water probe only shown. **(d)** For comparison, the network calculated by WaterFLAP for the less potent phenyl analog (N → C) **4d**.

**Figure 5:**
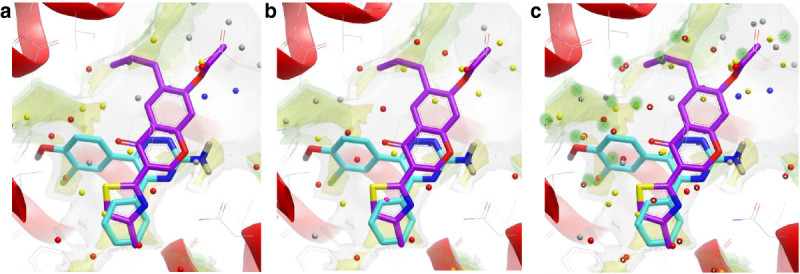
**Water networks from WaterMap and WaterFLAP for the pseudo-apo adenosine A**_**2A**_**receptor shown with two potent ligands. (a)** The WaterMap waters for the high resolution A_2A_ structure (PDB:4EIY) are colour coded as in Figure 1, with the ‘unhappy’ waters in red and yellow. **(b)** The WaterFLAP waters with the ‘unhappy’ waters coded in red then yellow. **(c)** Waters calculated by both WaterMap and WaterFLAP are shown, with WaterFLAP waters distinguished by a white circle. Also shown in a ‘glowing’ green are the crystallographic waters from the high resolution structure with ZM241385 (PDB:4EIY), that binds similar to the chromone (but does not have the propyl chain, see figure 10 in Congreve et al., 2011). The GRID lipophilic C1= map is contoured at -2.5 kcal/mol together with the water (OH2) probe in green at –6.0 kcal/mol to highlight the druggability and key binding regions of the binding pocket; CH3 methyl group probe shown in grey at 1 kcal/mol to define the pocket shape.

The availability of multiple diverse X-ray structures for the adenosine A_2A_ receptor in complex with antagonist or experimentally-enabled docking poses enables a broader view of the ligand-efficient binding modes. Figure 4a shows two potent leads from the triazine and chromone series bound, together with the GRID lipophilic hotspots. It is clear that between the two series all the lipophilic hotspots are used, in different combinations. The WaterMap waters for the pseudo-apo structure are shown in Figure 5a and the WaterFLAP waters in Figure 5b. The water networks were calculated for the pseudo-apo structure to highlight waters displaces by the ligand. With WaterMap only the pseudo-apo structure was used as this gave best results, but as shown in Figure 4b-c-d WaterFLAP was also used robustly for the A_2A_ triazine complexes, showing clearly the difference in networks for the different potency ligands. The druggable subpocket with a lipophilic hotspot used by the propyl group of the chromone ligand was best distinguished by WaterFLAP, that predicted the region to be occupied by yellow and red ‘unhappy’ waters; WaterMap predicted one ‘happy’ (between 1^st^ and 2^nd^ carbon of the propyl group) and one ‘unhappy’ more distant water. This region is less evident in the lower resolution ZM214385 structure (PDB:3EML, one yellow water, figure 10 in Congreve et al., 2011), the triazine 4g (PDB:3UZA, one yellow water, Figure 1,^2012^); and the 4e triazine complex (PBD:3UZC, both grey, Figure 2 in Andrews SP, Mason JS, Hurrell E, Congreve M: Structure-Based Drug Design of Chromone Antagonists of the Adenosine A2A Receptor. Med Chem Comm, accepted)(WaterMap calculations). The general consensus in both the position and energy of waters in the site from the very different approaches WaterMap and WaterFLAP can be seen in Figure 5c, where both are shown. Also included in Figure 5c are the binding site crystallographic waters from the high resolution ZM241385 structure, which encouragingly were all mapped in a similar region by the computational methods, WaterFLAP finding all of them. Note that a simple approach using an MD optimization of the protein-ligand-water network using a default equilibrated box of TIP3P waters (i.e. skipping the initial water placement based on GRID) does not work, resulting in a completely ‘dewetted’ binding site. It is important to be able to predict the water network for GPCR ligand complex structures as the high resolution required to accurately identify crystallographic waters is rarely achievable with GPCR structures; experience with GPCR StaR structures at Heptares has shown though that the ligand electron density however is well defined even at lower overall resolutions. Irrespective of the source of the waters, having a computational estimation of the relative free energies of the waters is a very useful addition.

The WaterFLAP approach used (GRID-based water placement followed by MD optimization) is good at producing complete water networks for these ligand complexes with explicit optimised H-bonding networks, and this is shown in Figure 4b, 4c and 4d for the X-ray complexes of the triazines 4e, 4g and 4d. The role of the pyridine nitrogen in producing a good network can be seen, the positive effect of the pyridine N on binding not being evident by looking at only ligand-receptor complexes. The less optimal water network deep in the binding site is also shown for the phenyl analog in Figure 4d. Water network energetics were shown (Bortolato et al., 2013) for a series of triazine analogues for the A_2A_ receptor to be related to residence times, with a change of off-rate from 0 s to 87 s to 990 s for the unsubstituted phenyl to dimethyl pyridine (4e) to chlorophenol (4g) compounds. In particular ‘unhappy’ waters trapped between the ligand and the protein can be qualitatively linked to the decrease of the small molecules residence time.

### Structural rationalization of a ‘magic methyl’ in A_2A_ chromone antagonist ligands

Chromone12 (Langmead et al., 2012) is known to bind potently with a pK_i_ of 8.5 to the A_2A_ receptor, while the des-methyl derivative binds with a significantly 33x lower affinity (Andrews SP, Mason JS, Hurrell E, Congreve M: Structure-Based Drug Design of Chromone Antagonists of the Adenosine A2A Receptor. Med Chem Comm, accepted). This is a similar difference in activity to the ‘magic methyl’ recently published (Lunn et al., 2011) for a series of opioid antagonists but with no structural rationalization.

**Figure 6:**
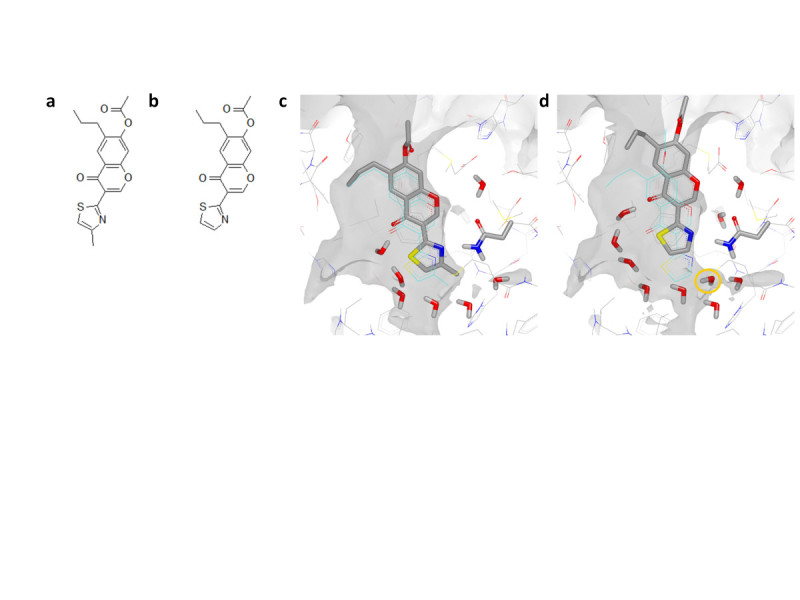
**WaterFLAP predictions for the A**_**2A**_**chromone ligands chromone12 and the des-methyl analog.** (**a-b)** 2D ligand structures of chromone 12 and the des-methyl analog. **(c)** The potent chromone12 ligand (pKi 8.5) is stable under 100 ps of MD simulation, with the methyl group remaining anchored in the lipophilic pocket bounded by Met177 and Leu249. The starting position of the ligand is shown in cyan wireframe, while the final position is shown in grey stick. The ligand retains a hydrogen bond to the key residue Asn253, shown in stick, and MD waters in the lower half of the binding site are shown. **(d)** Removal of the methyl leaves a vacuum in the structure, that is filled by a water molecule when the ligand moves up during MD simulation, resulting in a weakened interaction with Asn253 and ‘unhappy’ water(s) now filling this lipophilic region (highlighted in the orange circle; see Figure 5 where there is an ‘unhappy’ water in this region in the pseudo-apo structure). This compound has 33x reduced affinity.

To explain the significantly decreased affinity of the des-methyl compound we used water network placement and MD simulation to measure the effect of the ligand methyl on the binding and water network (Figure 6). The binding mode of chromone compound 12, consistent with the Biophysical Mapping data (Langmead et al., 2012) and X-ray structural data (unpublished data), has the thiazole nitrogen hydrogen bonded to the key residue Asn253^6.55^. This places the methyl group into a small hydrophobic pocket bounded by Met177 and Leu249. Both the WaterMap and WaterFLAP analyses place a very ‘unhappy’ water in the position occupied by the ligand methyl. The significant affinity gain by having this substituent can thus be understood in terms of the favourable lipophilic/hydrophobic interaction coupled to the free energy gain from displacement of the ‘unhappy’ water.

To structurally understand the effect on binding of removing this methyl group, a water network was generated with WaterFLAP and a longer, unconstrained MD run. Removing the methyl without adjusting the ligand placement would leave a vacuum in the protein structure that is not large enough for a water molecule and for this reason we postulated that the des-methyl ligand would bind higher in the binding site (i.e. closer to the extracellular side) thereby weakening the hydrogen bond to the critical Asn253^6.55^, and allowing at least one ‘unhappy’ water back into the lipophilic pocket. Indeed, after a 100 ps MD simulation the des-methyl derivative moves up, weakening the hydrogen bond, and water molecules move back, able to hydrogen bond to the surrounding water network (Figure 6f). The water positions are similar to those calculated for this region in the apo structure (Figure 5) that were scored as ‘unhappy’; the proximity of the ligand carbon atoms in the complex trapping the waters would be expected to make these waters even more ‘unhappy’. As a control, in the same MD simulation on the potent chromone12 the ligand moves very little after 100 ps, maintaining a hydrogen bond with Asn253^6.55^ (Figure 6c). This postulated change in position of the core binding group in the chromone series when the methyl is removed is another example of the danger of transferring SAR between compounds assuming a common binding position of the core group.

### Structural interpretation of mutational data in GPCR binding sites

Biophysical Mapping (BPM) is an experimental approach used to map binding site interactions with a ligand of interest (Zhukov et al., 2011). In the BPM approach additional single mutations are added to the StaR at positions that could be involved in small molecule interactions. The StaR and the panel of binding site mutants are captured onto Biacore chips to enable characterization of the binding of small molecule ligands using surface plasmon resonance (SPR) measurement. A matrix of binding data for a set of ligands versus each active site mutation is then generated, providing specific affinity and kinetic information (K_D_, k_on_, and k_off_) of receptor-ligand interactions. This data set, in combination with molecular modeling and docking, is used to map the small molecule binding site for every compound.

**Figure 7:**
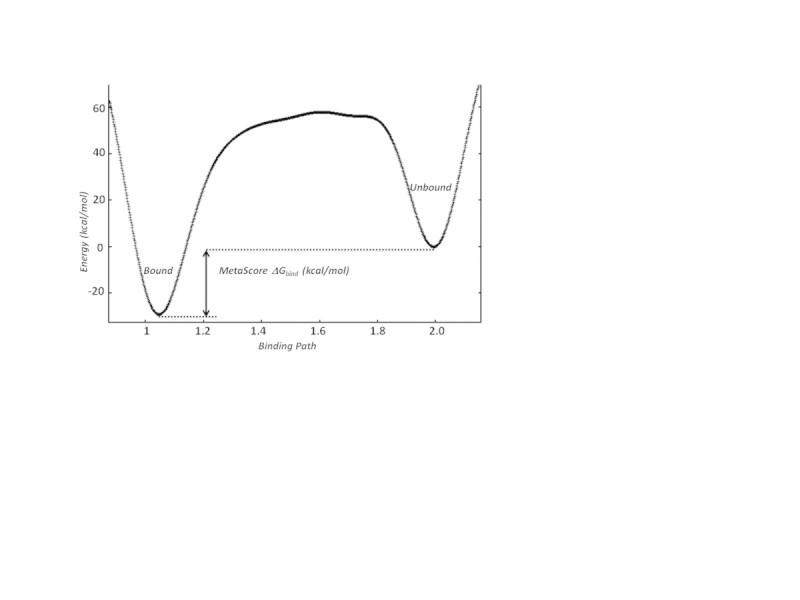
**Example free energy surface predicted by MetaScore for ligand binding.** The final MetaScore ΔG_bind_ for the small molecule is calculated as the energy difference between the bound state and the unbound state.

We developed a fast computational protocol to better understand *in silico* the BPM results based on a combination of enhanced sampling MD (MetaScore) and water network perturbation predictions (WaterFLAP). MetaScore uses adiabatically biased MD to evaluate a possible ligand binding path from the bulk solvent to the final crystallographic pose and metadynamics to evaluate the energy profile of the binding event (Figure 7). WaterFLAP allows the prediction of mutation effects on the water network in close proximity to the small molecule, and hence to the binding affinity.

**Figure 8:**
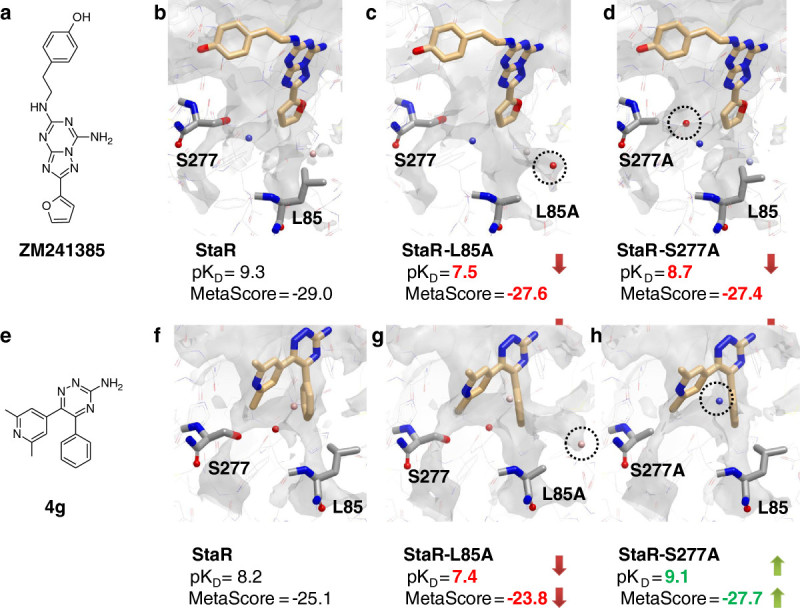
**WaterFLAP predictions for the ligands ZM241385 (top row) and 4g (bottom row).** Ligand 2D molecular structures are shown on the left **(a** and **e)**. For every protein-ligand complex the affinity (pK_D_) and the MetaScore ΔG_bind_ in kcal/mol is reported. The effect of the mutation decreasing or increasing the affinity compared to StaR is highlighted respectively in red and green. In the 3D figures **(b, c, d, f, g** and **h)**, ligands are shown in light orange stick representation, while residues 85 and 277 are shown in grey stick. Predicted water molecules are shown as sphere colour-coded by estimated free energy of interaction (using the GRID OH2 and CRY probes) from red (high energy, ‘unhappy’) to blue (low energy, ‘happy’). Only water molecules relevant to understand the BPM results are shown. Water molecule differences between the StaR and the protein mutants are highlighted by a black circle. The shape of the ligand binding site is represented using a solid grey surface created using the GRID C3 probe (carbon sp^3^) with a threshold energy value of 1 kcal/mol.

We applied the two novel *in silico* approaches to the adenosine A_2A_ receptor in complex with two small molecule antagonist: ZM241385 and 4g. We evaluated the effect of the mutations L85A^1.52^ and S277A^7.42^ on ligand binding. The predictions were compared to the experimental BPM results (Zhukov et al., 2011). For both ligands MetaScore was able to reproduce *in silico* the qualitative affinity (pK_D_) change (increase or decrease) resulting from the mutations (Figure 8) compared to the original StaR receptor. We used WaterFLAP to better understand the effect of perturbations in the receptor-ligand-water network as a consequence of the mutations, and to relate these to ligand affinity. Decrease of antagonist affinity seems related to additional ‘unhappy’ waters trapped between the ligand and the protein (Figure 8c-d-g), while increase of affinity is linked to the displacement of one ‘unhappy’ water (Figure 8h). While MetaScore can qualitatively predict the pK_D_ change resulting from mutations, WaterFLAP can give insight into the effect of the mutation even when the residue is not in direct contact with the ligand, through water network perturbation.

These preliminary results suggest that the combination of MetaScore and WaterFLAP can be an interesting computational protocol applicable to understand site directed mutation (SDM) data and to rescore docking poses. The approach is at the moment under testing and active development aiming to achieve a method accurate and fast enough to be usable in hit-to-lead or lead-optimization phases of drug discovery.

## Conclusions

Several recent advances in GPCR biophysical screening and X-ray crystallography allow for advanced structure-based drug design (SBDD), which we have termed ‘high end design’. These include: (1) The availability of X-ray structures of a diverse set of GPCRs in complex with multiple ligands, (2) StaR proteins, which allow among others fragment screening, Biophysical Mapping and binding kinetics and (3) computational analyses and design approaches that consider explicit waters and their calculated energies.

We consider the complete water network to be an essential third dimension in ligand design (after the protein and ligand structure), and this has been illustrated for several GPCR structures. This includes the estimation of relative water free energies, for both (pseudo)apo and ligand complex structures, that can be used for druggability, ligand binding (including selectivity) and binding kinetics estimations. It would be expected that the same approach could be successfully applied to enzyme targets, and this was the case for druggability in a previous paper (Mason et al., 2012). The ability to rapidly see explicit hydrogen bonded water networks, and to estimate free energy differences, for existing and proposed ligand modifications should not be underestimated. Linked to these calculations are the GRID energetic surveys of the site, that are found to be extremely useful in driving ligand design and evaluating docking poses. In particular the lipophilic hotspots from the C1= and the new CRY probe appear to be important in all the GPCR structures we have studied. The new software WaterFLAP, used in our protocol in conjunction with MD optimization, is showing great promise in providing an additional way to generate and score water networks for ligand-protein complexes, complementary to the established more computationally intensive and rigorous MD based approach WaterMap.

High end GPCR design has arrived!

## Authors’ original submitted files for images

Below are the links to the authors’ original submitted files for images. Authors’ original file for figure 1 Authors’ original file for figure 2 Authors’ original file for figure 3 Authors’ original file for figure 4 Authors’ original file for figure 5 Authors’ original file for figure 6 Authors’ original file for figure 7 Authors’ original file for figure 8

## Abbreviations

GPCR: G Protein-Coupled Receptors
A2A: Adenosine A_2A_ receptor
SBDD: Structure-based drug design
MIFs: Molecular interaction fields
MD: Molecular dynamics
ABMD: Adiabatic bias molecular dynamics
BPM: BioPhysical Mapping™
CCR5: C-C chemokine receptor type 5.
