# Supplementary figures and images for: High end GPCR design: crafted ligand design and druggability analysis using protein structure, lipophilic hotspots and explicit water networks
Source: In Silico Pharmacol. 2013 Dec 20;1:23. doi: 10.1186/2193-9616-1-23 (PMC4796210; doi:10.1186/2193-9616-1-23)

**a**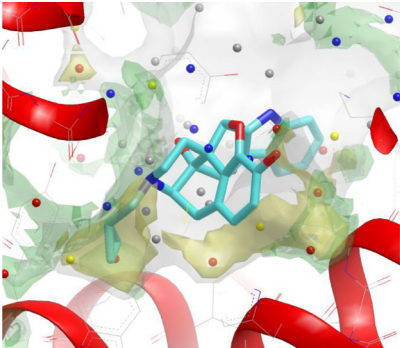**b**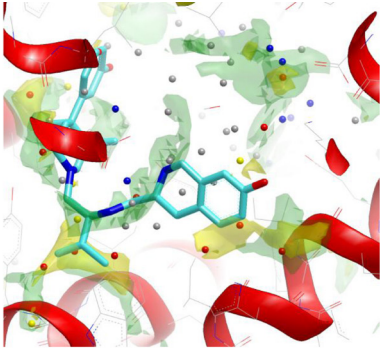**c**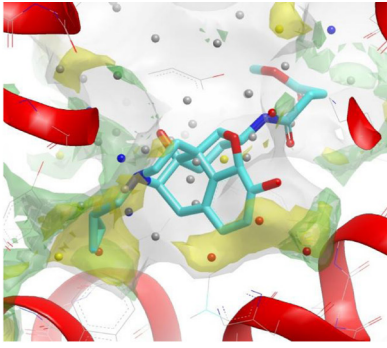

Supplement: Supplementary file 1 — Authors’ original file for figure 1 [file 40203_2013_25_MOESM1_ESM.pdf]

**a**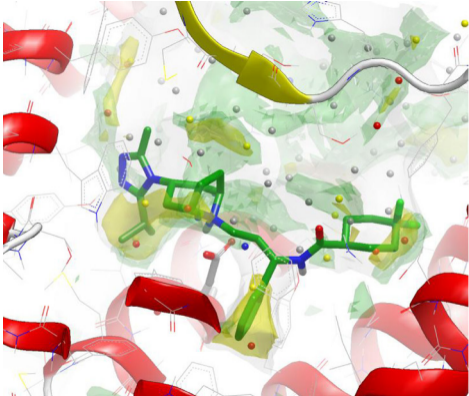**b**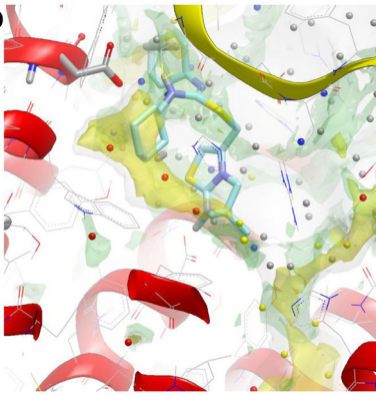**c**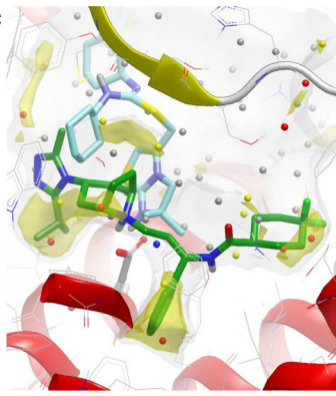

Supplement: Supplementary file 2 — Authors’ original file for figure 2 [file 40203_2013_25_MOESM2_ESM.pdf]

**a**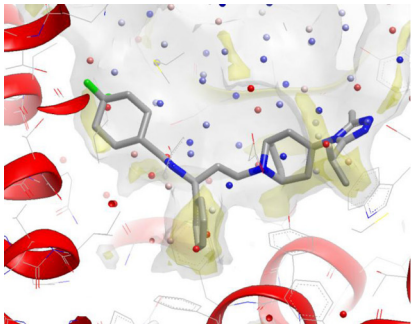**b**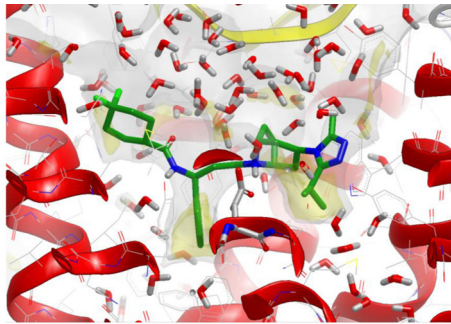

Supplement: Supplementary file 3 — Authors’ original file for figure 3 [file 40203_2013_25_MOESM3_ESM.pdf]

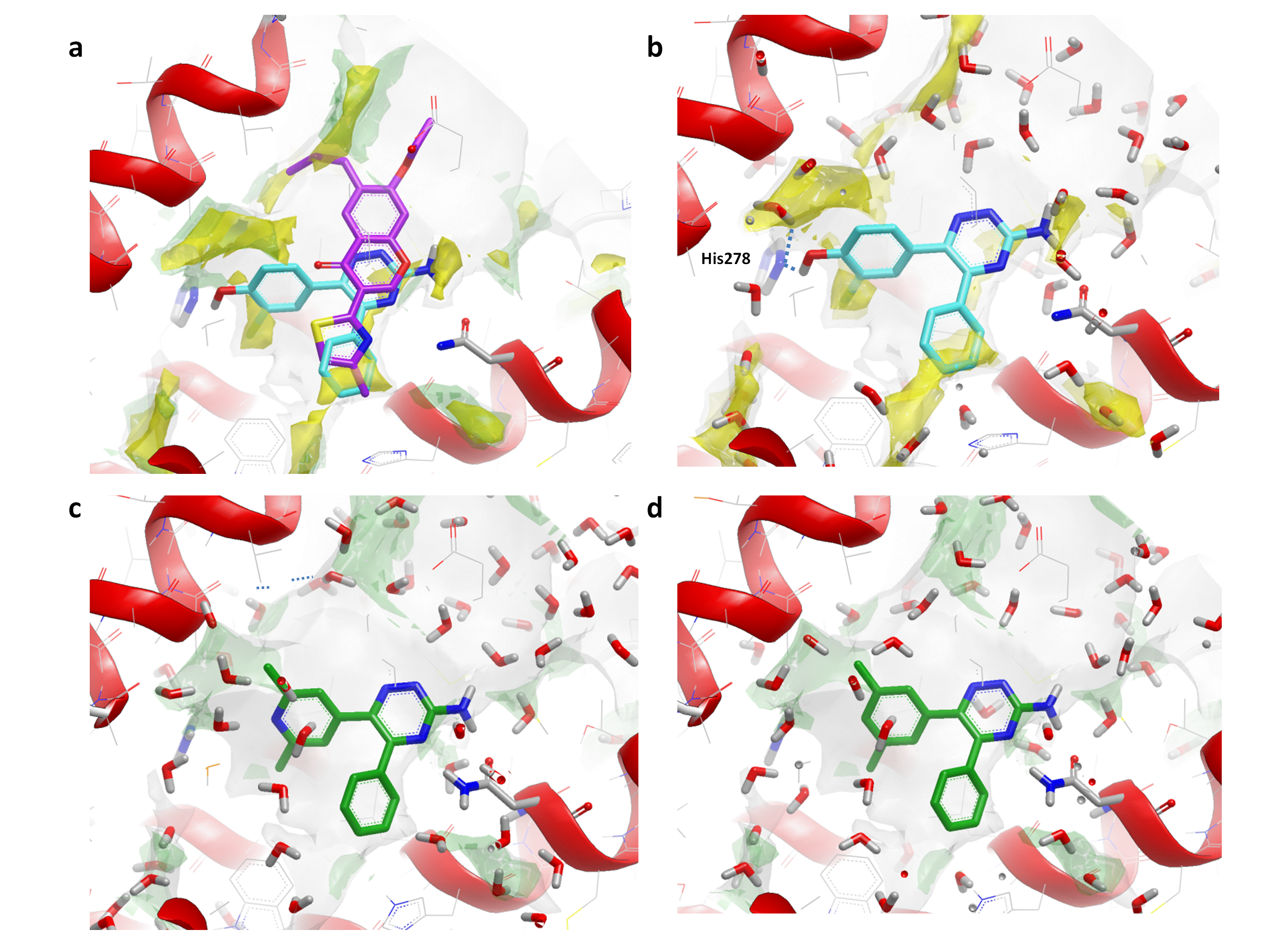

Supplement: Supplementary file 4 — Authors’ original file for figure 4 [file 40203_2013_25_MOESM4_ESM.gif]

**a**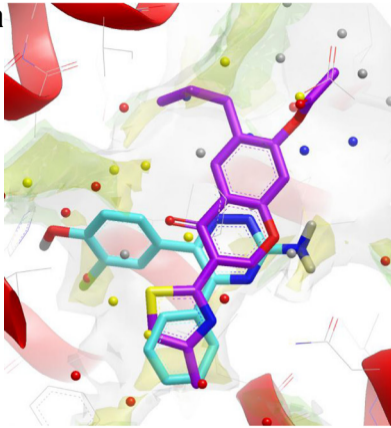**b**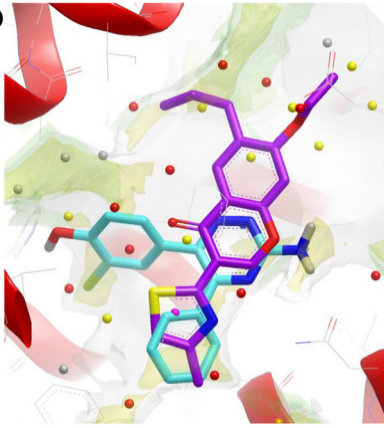**c**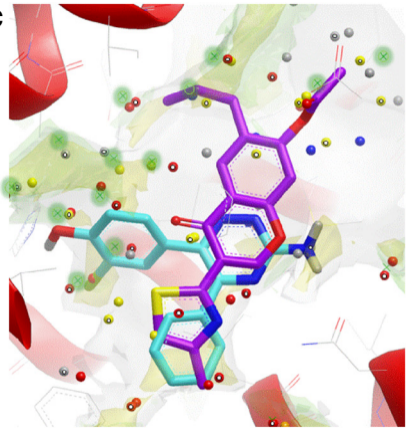

Supplement: Supplementary file 5 — Authors’ original file for figure 5 [file 40203_2013_25_MOESM5_ESM.pdf]

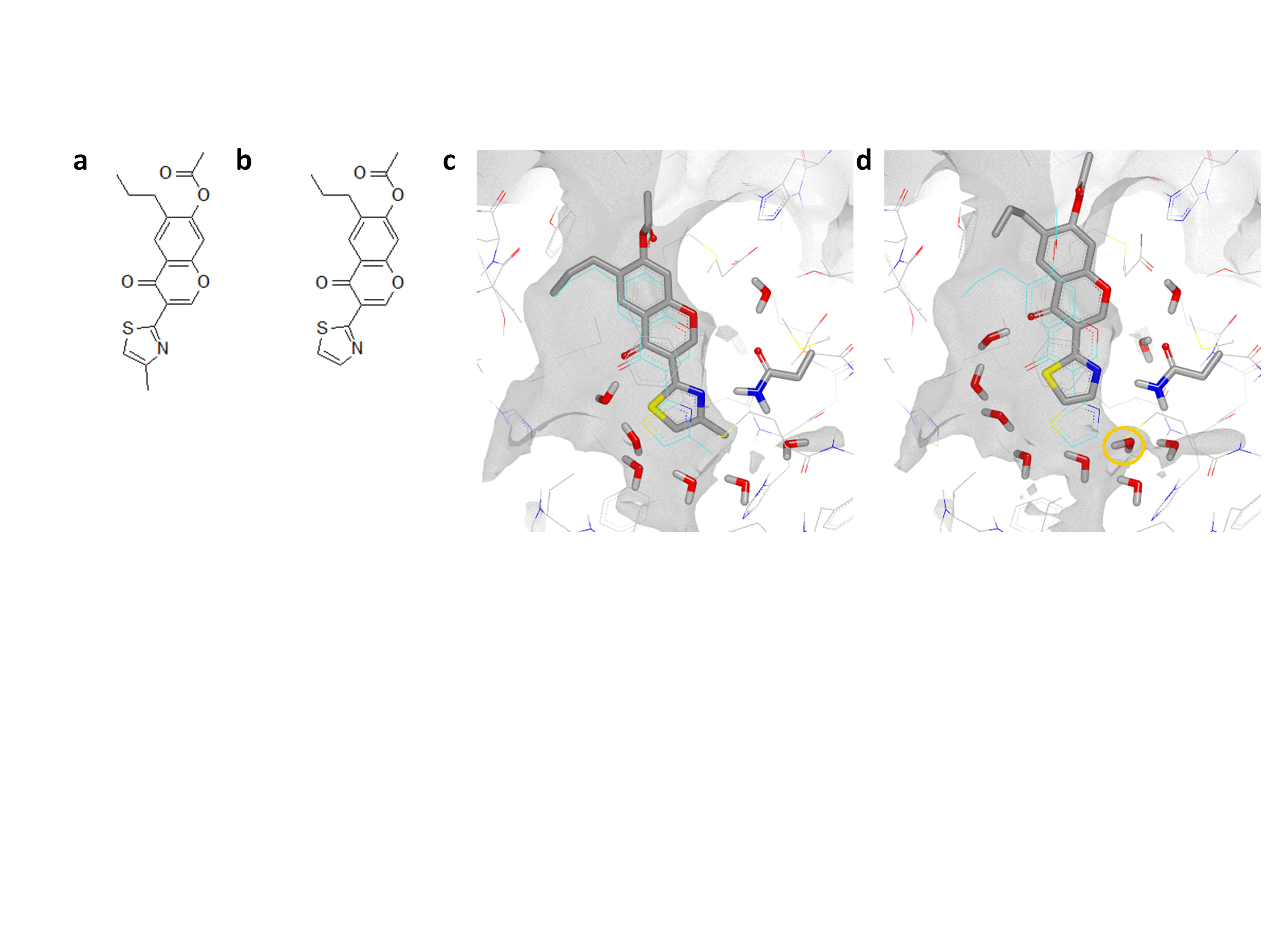

Supplement: Supplementary file 6 — Authors’ original file for figure 6 [file 40203_2013_25_MOESM6_ESM.gif]

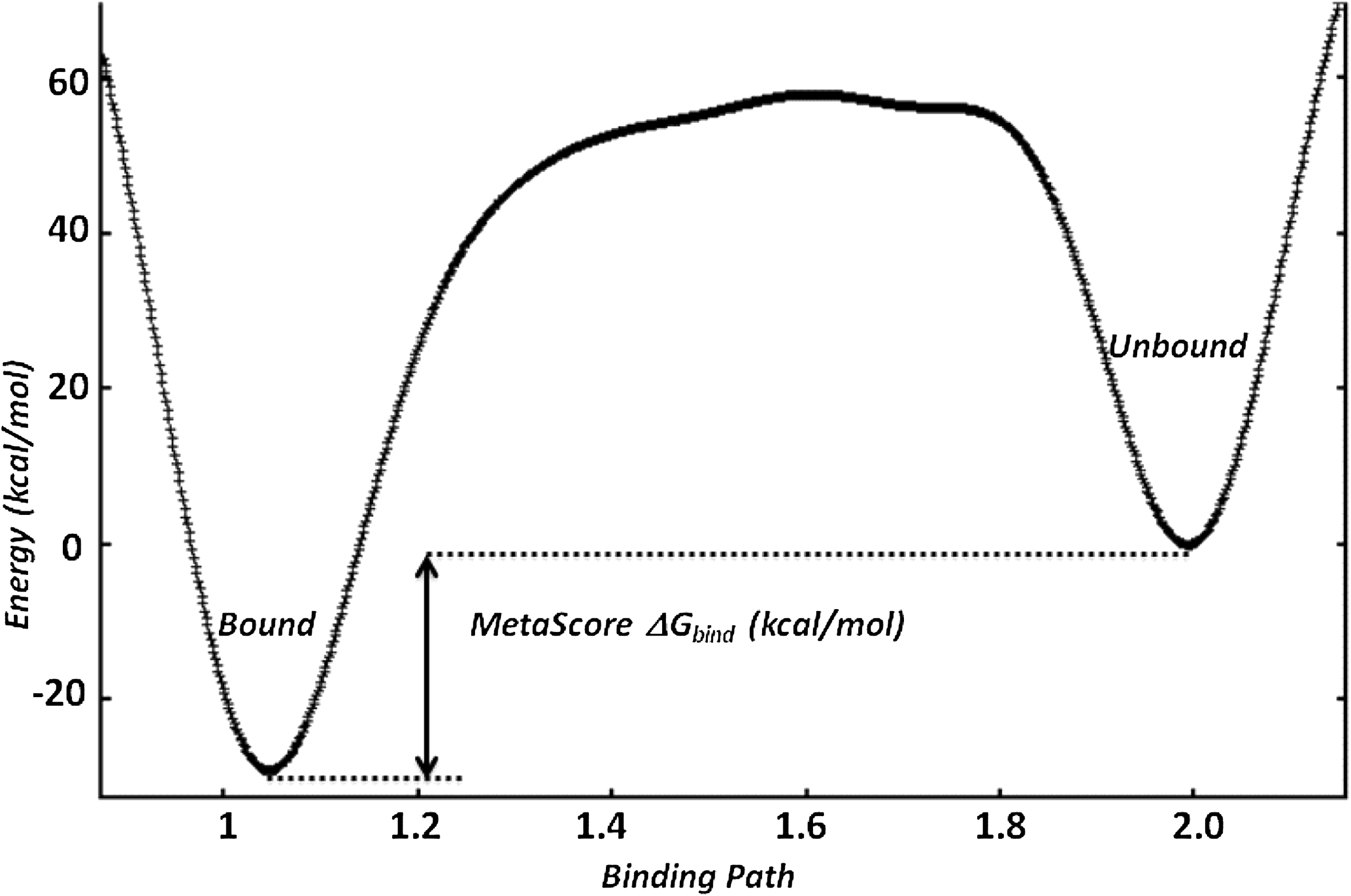

Supplement: Supplementary file 7 — Authors’ original file for figure 7 [file 40203_2013_25_MOESM7_ESM.tif]
