# Supplementary material for: High end GPCR design: crafted ligand design and druggability analysis using protein structure, lipophilic hotspots and explicit water networks
Source: In Silico Pharmacol. 2013 Dec 20;1:23. doi: 10.1186/2193-9616-1-23 (PMC4796210; doi:10.1186/2193-9616-1-23)
Supplement: Supplementary file 8 — Authors’ original file for figure 8 [file 40203_2013_25_MOESM8_ESM.pdf]

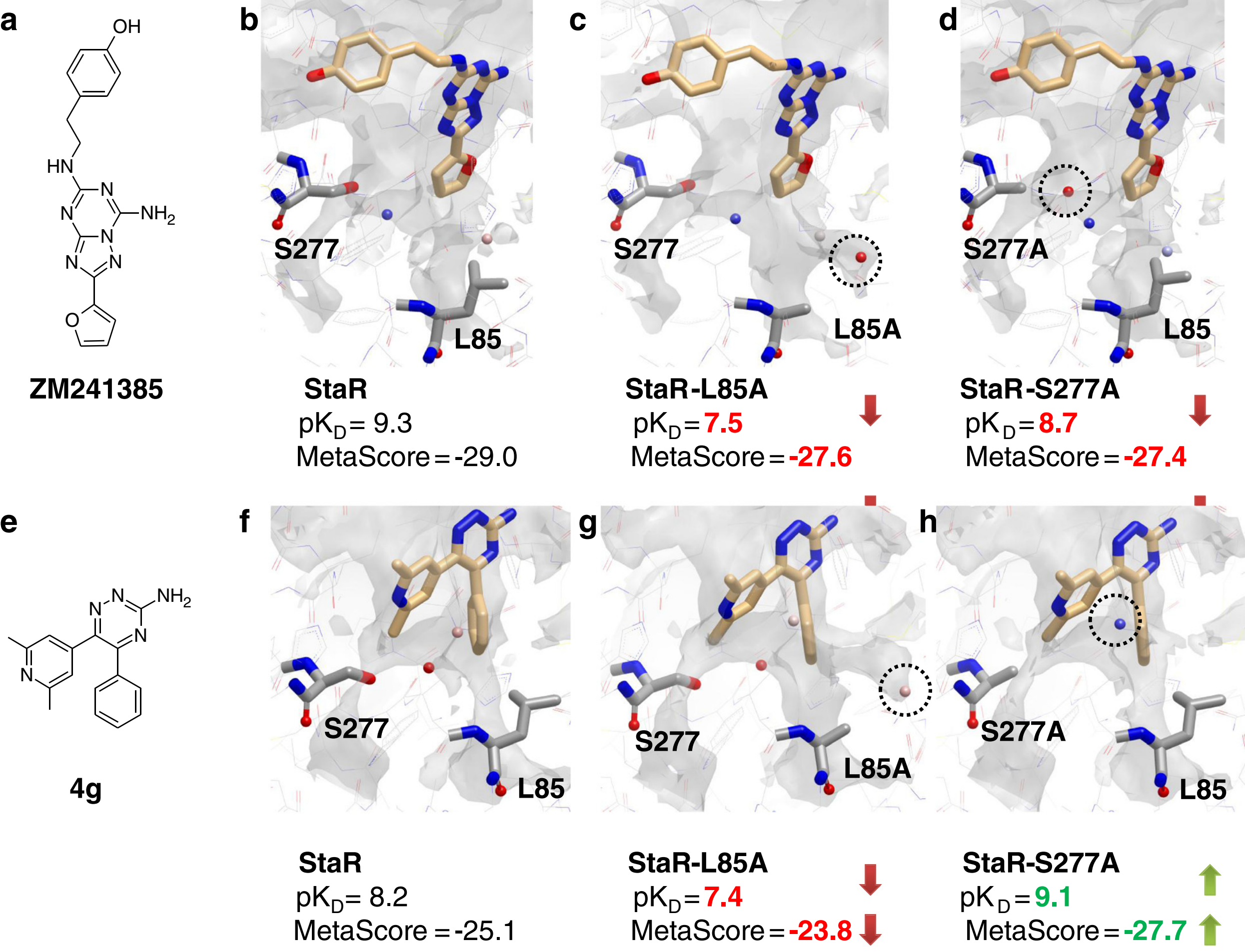
a
OH
HN
N
N
NH2
N
N
N
N
O
ZM241385
b
S277
L85
StaR
pKD= 9.3
MetaScore=-29.0
c
S277
L85A
StaR-L85A
pKD=7.5
MetaScore=-27.6
d
S277A
L85
StaR-S277A
pKD=8.7
MetaScore=-27.4
e
N
N
NH2
N
N
N
4g
f
S277
L85
StaR
pKD= 8.2
MetaScore=-25.1
g
S277
L85A
StaR-L85A
pKD=7.4
MetaScore=-23.8
h
S277A
L85
StaR-S277A
pKD=9.1
MetaScore=-27.7
